# Nonlinear Dynamic Analysis of a Piezoelectric Energy Harvester with Mechanical Plucking Mechanism

**DOI:** 10.3390/s23135978

**Published:** 2023-06-27

**Authors:** Jinhong Noh, Sungryong Bae, Yong-Jin Yoon, Pilkee Kim

**Affiliations:** 1 Department of Mechanical Engineering, Korea Advanced Institute of Science and Technology (KAIST), Daejeon 34141, Republic of Korea; jinhongnoh@kaist.ac.kr; 2 Department of Fire Protection and Disaster Management, Chosun University, Gwanju 61452, Republic of Korea; sbae@chosun.ac.kr; 3 School of Mechanical and Aerospace Engineering, Nanyang Technological University (NTU), Singapore 639798, Singapore; 4 School of Mechanical Design Engineering, College of Engineering, Jeonbuk National University, Jeonju-si 54896, Jeollabuk-do, Republic of Korea; 5 Eco-Friendly Machine Parts Design Research Center, Jeonbuk National University, Jeonju-si 54896, Republic of Korea

**Keywords:** piezoelectric energy harvester, mechanical plucking mechanism, differential transformation method, nonlinear dynamic analysis

## Abstract

In this study, we propose an analytical approach based on the modified differential transform method to investigate the dynamic behavior of a plucking energy harvester. The harvester consists of a piezoelectric cantilever oscillator and a rotating plectrum. The analytical approach provides a closed-form solution that helps determine the starting and ending points of the contact phase between the piezoelectric cantilever and the plectrum. This analytical approach is valuable for simulating complex dynamic interferences in multiple or periodic plucking processes. To evaluate the effects of plucking speed and overlap length of the plectrum on single and periodic plucking, a series of simulations were carried out. The output voltage of the piezoelectric energy harvester increases as the overlap length of the plectrum increases. On the other hand, increasing the plucking speed tends to amplify the magnitude of the contact force while reducing the duration of the contact phase. Therefore, it is crucial to optimize the plucking speed to achieve the maximum linear impulse. For periodic plucking, successful synchronization between the motions of the piezoelectric energy harvester and the rotating plectrum must occur within a limited contact zone. Otherwise, dynamic interferences often cause the plectrum to fail to pluck the energy harvester exactly within the contact zone. Additionally, reducing the plucking speed of the plectrum and increasing the overlap length would be more advantageous for successful periodic-plucking energy harvesting.

## 1. Introduction

Energy harvesting involves a series of processes aimed at extracting ambient energy, converting it into electrical energy, and utilizing or storing it to power micro-sensors or devices. Various ambient energy sources are available for energy harvesting, including sunlight, wind, heat, and vibration. Among these sources, mechanical vibration is commonly present in our daily lives. The energy conversion mechanisms for mechanical vibration include piezoelectric, electrostatic, and electromagnetic transductions [1,2,3], which are relatively straightforward compared to other energy sources. As a result, vibration energy harvesters [4,5,6,7,8,9,10,11,12,13,14,15,16,17,18,19,20,21,22,23,24,25,26,27] have been developed for diverse applications, including small-scale portable devices, MEMS sensors for the Internet of Things, and autonomous wireless sensor networks for smart environments.

In the early stages of development, vibration energy harvesters were primarily designed using linear electromechanical oscillators, which had a limited resonant frequency range. These linear resonant-type harvesters were only capable of generating high energy within their narrow resonant frequency band. Consequently, they proved to be unsuitable for complex ambient vibration sources that involved multiple frequency components and/or time-varying frequency components [4,5].

Extensive research efforts have been dedicated to expanding the frequency bandwidth of vibration energy harvesters. To achieve this, researchers have proposed and developed various energy harvesting structures, employing different technologies such as multi-modal oscillators or oscillator arrays [6,7,8], frequency-tunable oscillators [9,10], nonlinear monostable oscillators [11,12,13], and multi-stable oscillators [14,15,16,17,18,19,20,21,22,23,24]. However, the utilization of multi-modal oscillators or oscillator arrays often resulted in larger and more complex structures for energy harvesting. Nonlinear monostable or multi-stable oscillators showed promise for broadband energy harvesting, but the presence of hysteresis due to multiple solutions often resulted in decreased energy harvesting efficiency. Conversely, an alternative approach known as frequency-up conversion has emerged for harvesting energy from low-frequency ambient vibrations [25,26,27,28,29,30]. This technique involves utilizing mechanical/magnetic impact or a plucking force to stimulate the high-frequency natural vibration of the energy-harvesting oscillator, which can then be converted into electrical energy.

Mechanical plucking energy harvesters have been developed to extract energy from the periodic motions of rotor blades or rotating plectra in various applications, such as wind turbines, human knee joints, and automobile wheels [18,19,20,21,22,23]. The fundamental design of mechanical plucking energy harvesters consists of an energy-harvesting oscillator and an external plectrum. When the plectrum impacts the energy-harvesting oscillator that has a relatively high natural frequency, it imparts a linear impulse to the oscillator. Subsequently, the oscillator undergoes free vibrations, which are then converted into electrical energy. The main challenges in designing such mechanical plucking energy harvesters arise from the contact mechanism between the energy-harvesting oscillator and the external plectrum, involving complex dynamic interactions [31,32,33,34,35,36,37]. Mathematical models based on Hertzian contact theory [36,37,38,39] and methodologies utilizing finite element analysis or other numerical methods have been employed to predict the complex dynamic behaviors of the mechanical plucking energy harvesters, including strong dynamic interferences and multiple or periodic plucking effects.

In this study, we propose an analytical approach based on the modified differential transform method to investigate the dynamic behaviors of a mechanical plucking energy harvester, which comprises a piezoelectric cantilever oscillator and a rotating plectrum. The analytical approach provides a closed-form solution that facilitates the determination of the starting and ending points of the contact phase between the piezoelectric cantilever and the plectrum. This analytical approach is valuable for simulating complex dynamic interferences in multiple or periodic plucking processes. We conduct a series of simulations to evaluate the effects of plucking speed and overlap length of the plectrum on single or periodic plucking, and we discuss the results.

## 2. Mathematical Model

### 2.1. Piezoelectric Energy Harvester with a Mechanical Plucking Mechanism

**Figure 1:**
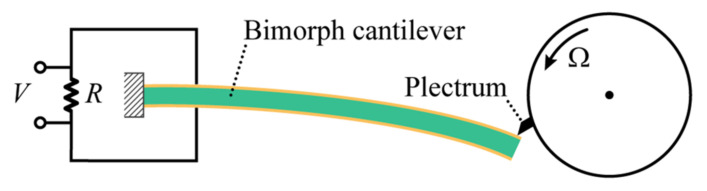
Schematic of a piezoelectric energy harvester with a mechanical plucking mechanism.

Figure 1 shows the schematic diagram of a piezoelectric energy harvester with a mechanical plucking mechanism. The harvester consists of a piezoelectric bimorph cantilever beam and a rotating plectrum. The bimorph cantilever beam comprises a stainless-steel substrate partially covered by two identical piezoelectric films which are polarized oppositely in the vertical direction. The natural frequency of the bimorph cantilever beam oscillator can be adjusted by altering the thickness of the stainless-steel substrate while keeping the dimensions of the piezoelectric layers unchanged. Both major surfaces of each piezoelectric layer are fully coated with conductive electrodes. These two piezoelectric layers are connected in series to an external load resistance *R*. Table 1 provides the geometric parameters and material properties of a typical piezoelectric bimorph cantilever.

When the rotating plectrum plucks the tip of the bimorph cantilever, the cantilever structure is deflected, and the resulting mechanical strain in the piezoelectric materials is converted into the electrical current that flows through the external load resistance. Through this energy conversion mechanism, the piezoelectric cantilever energy harvester can extract useful electrical energy from rotational motions, such as individuals passing through turnstile gates, rotating vehicle wheels, and wind turbines, to power autonomous micro-sensors or devices.

### 2.2. Electromechanical Duffing Oscillator Model of the Mechanical Plucking Energy Harvester

The piezoelectric bimorph cantilever beam is modeled based on the Euler–Bernoulli beam theory and linear piezoelectricity. The Kelvin–Voigt damping model is employed to account for the material damping effect of the cantilever beam. Firstly, the governing field equations and boundary conditions are derived using D’Alembert’s principle and the moment balance method [13]. Following a series of derivation processes, including modal analysis, discretization process, and single-mode approximation [13,14,15], the differential equations of motion for the mechanical plucking energy harvester and the initial conditions can be obtained as follows:

(1) $$\ddot{\tilde{w}}+2\zeta \omega_{n}\dot{\tilde{w}}+\omega_{n}^{2}\tilde{w}+\beta {\tilde{w}}^{3}-\theta \tilde{V}=\alpha f_{p},$$

(2) $$C_{p}\dot{\tilde{V}}+\frac{1}{R}\tilde{V}+\theta \dot{\tilde{w}}=0,$$

(3) $$\tilde{w}(0)={\bar{w}}_{0},\dot{\tilde{w}}(0)={\bar{v}}_{0},\tilde{V}(0)={\bar{V}}_{0},$$

where the dot over the symbols indicates the time derivative; $\tilde{w}$ is the tip displacement of the cantilever beam; ζ and $\omega_{n}$ are the natural angular frequency and damping ratio, respectively; β is the coefficient of the cubic nonlinear term; θ is the electromechanical coupling constant; $\tilde{V}$ is the output voltage; R and $C_{p}$ are the external load resistance and equivalent capacitance; and $\alpha f_{p}$ is the generalized plucking force. In Equation (1), the cubic nonlinear term is added to describe some nonlinear effects such as nonlinear magnetic interactions [39] and structural nonlinearity [13]. The derivation process for the equivalent circuit equation, given by Equation (2), is provided in Appendix A. Additionally, to validate the oscillator model, a comparison is made between the numerical results obtained using Equations (1) and (2) and the experimental results reported in Ref. [37], which are provided in Appendix B.

For generality, introducing the following dimensionless variables and parameters to Equations (1)–(3),

(4) $$x=\frac{\tilde{w}}{W},\;y=\frac{\tilde{V}}{V},\;t=\frac{\tilde{t}}{T}=\omega_{n}t,\;f=\frac{\alpha }{W\omega_{n}^{2}}f_{p},$$

(5) $$\eta =2\zeta ,\gamma =\frac{\beta }{\omega_{n}^{2}}W^{2},\;V=\frac{\omega_{n}^{2}}{\theta }W,\;\rho =\frac{1}{RC_{p}\omega_{n}},\;\theta_{e}=\frac{2\theta^{2}}{C_{p}\omega_{n}^{2}},$$

the dimensionless electromechanical oscillator model for the plucking energy harvester and the initial conditions are obtained in the form:

(6) $$\ddot{x}+\eta \dot{x}+x+\gamma x^{3}-y=f,$$

(7) $$\dot{y}+\rho y+\theta_{e}\dot{x}=0,$$

(8) $$x(0)=x_{0},\;\dot{x}(0)=v_{0},\;y(0)=y_{0}.$$

Note that the dimensionless mathematical model is used for all the dynamic simulations conducted in this study. Henceforth, unless specified otherwise, the variables and parameters will be treated as dimensionless.

In general, the plucking process consists of two distinct phases: the contact phase, during which the tips of the bimorph cantilever beam and the plectrum remain in contact, and the release phase, in which the beam undergoes free vibration after the separation from the plectrum. Although the contact phase has a much shorter duration compared to the release phase, it holds greater significance as it provides the kinetic energy required to initiate the subsequent free vibration of the energy harvester. The contact phase involves complex dynamic interactions between the beam and plectrum tips, encompassing sliding, shock, and vibration [38]. Furthermore, it is well-known that when the plucking speed is small (quasi-static plucking process), excessive surface friction significantly impacts the dynamic interaction, giving rise to complicated effects such as the stick-slip phenomenon. In this study, we focus on relatively high plucking speeds (dynamic plucking process), assuming the negligible influence of surface friction during the contact phase.

In the dynamic plucking process, the contact period is very short, and no plastic deformation occurs. This implies that the contact force can be considered as a spring force, dependent on local elastic deformations. Under these assumptions, the plucking force, denoted as *f* in Equation (6), is modeled based on the Hertzian contact theory [37,39]. Additionally, assuming a linear relationship between the contact force and the indentation, the plucking force can be expressed as follows:

(9) $$f=\left\{\begin{array}{cc} \kappa \delta =\kappa (u-x), & \delta \geq 0\\ 0, & \delta <0 \end{array}\right.\;\mathrm{where}\;u=R\sin(\omega t+\phi_{0}),$$

where κ is the linear contact stiffness; δ is the indentation; u is the vertical displacement of the plectrum; R is the radius of curvature of the circular motion of the plectrum; and ω and $\phi_{0}$ are the angular frequency and phase angle, respectively. In Equation (9), δ≥0 for the contact phase, whereas δ<0 for the release phase after the loss of contact.

The mechanical linear impulse, which is the integral of the plucking force over the time interval of the contact phase, determines the change in the linear momentum of the cantilever beam and initiates its free vibration. This linear impulse depends on both the duration of the contact and the contact area. The contact duration, denoted as Δt, is measured from the initial contact time, $t_{0}$, to the separation time, $t_{s}$, and by setting $t_{0}=0$, it becomes equivalent to the separation time itself, i.e., $\Delta t=t_{s}$. If the overlap length is very small, the geometric separation distance can be approximated as equal to the overlap length between the cantilever beam and the plectrum at a trivial position, as shown in Figure 1. Therefore, the contact duration or the separation time can be evaluated as follows:

(10) $$\Delta t=t_{s}\approx \frac{w_{d}}{v_{p}}\;\mathrm{and}\;v_{p}=\left|\dot{u}\right|=R\omega$$

where $w_{d}$ is the overlap length and $v_{p}$ is the plucking speed. 

In previous studies, Equation (10) was used to determine the end of the contact phase in the plucking process. However, it is important to note that the separation time obtained from the geometric configuration may or may not coincide with the actual end of the contact phase. This is because contact loss can occur within the separation distance due to excessive relative motion between the cantilever beam and the plectrum. In this study, for all simulations conducted, it is determined whether contact loss occurs within the separation distance. To accomplish this, the analytical solution of the initial value problem described by Equations (6)–(8) is obtained and used to determine the condition of zero indentation, which represents the actual end of the contact phase prior to the separation distance. 

## 3. Methodology

### 3.1. Differential Transform Method

In this study, we utilize an analytical technique known as the differential transformation method (DTM) to derive a closed-form approximate solution of the nonlinear electromechanical model presented in the previous section for the mechanically plucked piezoelectric energy harvester.

The differential transformation of an analytical function f(t) at $t=t_{0}$, $f(t)\to F\left(k\right)$, is defined as

(11) $$F\left(k\right)=\frac{1}{k!}{\left.\frac{d^{k}f\left(t\right)}{dt^{k}}\right|}_{t=t_{0}}.$$

The differential inverse transformation $F\left(k\right)\to f(t)$ can be performed as follows:

(12) $$f\left(t\right)=\sum_{k=0}^{\infty }F\left(k\right)t^{k}=\sum_{k=0}^{\infty }\frac{1}{k!}{\left.\frac{d^{k}f\left(t\right)}{dt^{k}}\right|}_{t=t_{0}}t^{k}.$$

As presented in Equations (11) and (12), the transformed function F(k) is a sort of the coefficient of power series of the original function f(t). The fundamental operations of the DTM are provided in Table 2. Refer to References [40,41] for the detailed derivation of the fundamental DTM operations.

To solve the initial value problem of the Duffing oscillator forced by mechanical plucking, we apply the differential transformation method (DTM), at t=0, to the initial conditions and the system of ordinary differential equations given in Equations (6)–(8), which yields the transformed initial conditions

(13) $$X\left(0\right)=x_{0},\;X\left(1\right)=v_{0},\;Y\left(0\right)=y_{0}.$$

and the transformed system of equations

(14) $$\begin{array}{l} \left(k+1\right)\left(k+2\right)X\left(k+2\right)=-\eta \left(k+1\right)X\left(k+1\right)-X\left(k\right)+Y\left(k\right)\\ \;\;\;-\gamma \sum_{k_{2}=0}^{k}\sum_{k_{1}=0}^{k_{2}}X\left(k_{1}\right)X\left(k_{2}-k_{1}\right)X\left(k-k_{2}\right)+F\left(k\right),\;k=0,1,2,\cdots , \end{array}$$

(15) $$\left(k+1\right)Y\left(k+1\right)=-\rho Y\left(k\right)-\theta_{e}\left(k+1\right)X\left(k+1\right),\;k=0,1,2,\cdots ,$$

where

(16) $$F\left(k\right)=\left\{\begin{array}{ll} \kappa \left(U\left(k\right)-X\left(k\right)\right) & \;\mathrm{for}\;\delta \geq 0\\ 0 & \;\mathrm{for}\;\delta <0 \end{array}\right..$$

As shown in Equations (14) and (15), the DTM transforms the original differential equations into a system of algebraic equations involving the unknowns X(k) and Y(k), which are the transformed functions of displacement x(t) and voltage y(t), respectively. Specifically, Equations (14) and (15) form a recursive system of algebraic equations that allow us to determine the solution by substituting the values of X(k), X(k+1), and Y(k) into the equations. This process begins with the transformed initial conditions, for k=0, given in Equation (13).

Finally, using the differential inverse transformation, we obtain the approximate solution functions x(t) and y(t) for the initial-value problem in the following forms of truncated Maclaurin series up to the *N*th order:

(17) $$x\left(t\right)=\sum_{k=0}^{N}X\left(k\right)t^{k}\;\mathrm{and}\;y\left(t\right)=\sum_{k=0}^{N}Y\left(k\right)t^{k}.$$

Note that the solution functions are locally analytic at t=0, allowing them to be approximated by truncated power series centered around the initial point. The approximate solutions obtained from the DTM demonstrate improved convergence to the exact solution with reduced error, but this convergence is guaranteed only in the vicinity of the initial point. To expand the region of convergence beyond this limited range, we will apply the Laplace–Padé resummation method to the truncated series solutions in the next section.

### 3.2. Laplace–Padé Resummation Method

By applying the Laplace transform with respect to *t* to the truncated power series solutions given in Equation (17), we obtain the transformed solution functions in terms of *s*:

(18) $$L\left[x(t)\right](s)=\sum_{k=0}^{N}\frac{X\left(k\right)}{k!}s^{-(k+1)}\;\mathrm{and}\;L\left[y(t)\right](s)=\sum_{k=0}^{N}\frac{Y\left(k\right)}{k!}s^{-(k+1)},$$

where L denote the Laplace transform. 

The Padé approximants for the solution functions given in Equation (18) are then calculated. These approximants are known to provide the best approximation using a rational function of a specific order [42,43]. The Padé approximation is applied to obtain the solutions for displacement and voltage, which are given as follows:

(19) $$L\left[x(t)\right](s)\approx P_{x}^{L/M}(s)=\frac{\sum_{n=0}^{L}a_{n}s^{-n}}{\sum_{n=0}^{M}b_{n}s^{-n}}\;\mathrm{or}\;\sum_{k=0}^{N}\sum_{n=0}^{M}\frac{X\left(k\right)}{k!}b_{n}s^{-(k+n+1)}-\sum_{n=0}^{L}a_{n}s^{-n}\approx 0,$$

(20) $$L\left[y(t)\right](s)\approx P_{y}^{L/M}(s)=\frac{\sum_{n=0}^{L}c_{n}s^{-n}}{\sum_{n=0}^{M}d_{n}s^{-n}}\;\mathrm{or}\;\sum_{k=0}^{N}\sum_{n=0}^{M}\frac{Y\left(k\right)}{k!}d_{n}s^{-(k+n+1)}-\sum_{n=0}^{L}c_{n}s^{-n}\approx 0,$$

where L and M are the upper bounds of summations, $b_{0}$ is set to be zero for normalization, $a_{n}$ and $c_{n}$ are the L+1 numerator coefficients of the displacement and voltage solutions, respectively, and $b_{n}$ and $d_{n}$ are the M denominator coefficients. In Equations (19) and (20), by equating the coefficients of like powers of *s*, we obtain a linear system of L+M+1 algebraic equations for the unknowns $a_{n}$ and $b_{n}$, which can be solved. 

Finally, we apply the inverse Laplace transformation with respect to s to obtain the approximate solutions in the following forms:

(21) $$x(t)\approx L^{-1}\left[P_{x}^{L/M}(s)\right](t)\;\mathrm{and}\;y(t)\approx L^{-1}\left[P_{y}^{L/M}(s)\right](t),$$

Note that the numbers of the coefficients, L and M, can be arbitrarily chosen within the range of L+M+1≤N+1. It is known that the error of the Padé approximation is minimized when L=M+1 or L=M for a fixed value of L+M+1. The Padé approximants mentioned above help expand or improve the region of convergence of the series solutions and provide better approximations compared to the truncated Taylor series solutions (Equation (17)) which have limited convergence and are only accurate in the vicinity of the initial point.

### 3.3. Numerical Simulation

In the previous section, the DTM was enhanced by incorporating the Laplace–Padé resummation method to improve the accuracy of the approximate solution functions. These modified DTM (MDTM) solutions are then compared with numerical results for validation purposes. For this purpose, a series of numerical simulations are conducted.

By introducing the state vector $x=\left[x_{1},x_{2},x_{3}\right]=\left[\dot{x},x,y\right]$ into Equations (6)–(8), the governing differential equations are reformulated into a system of first-order differential equations in the following vector form:

(22) $$\dot{x}=f(x,t)=\left[\begin{array}{c} -\eta x_{1}-x_{2}-\gamma x_{2}^{3}+x_{3}+\kappa (R\sin(\omega t+\phi_{0})-x_{2})\\ x_{1}\\ -\rho x_{3}-\theta_{e}x_{1} \end{array}\right],$$

The transient response of the piezoelectric energy harvester is obtained by numerically integrating Equation (22) using the Runge–Kutta method with the ode45 function in MATLAB. The baseline values of the dimensionless parameters are given as η=0.0146, γ=0.00, κ=316.23, R=300, ω=0.1, w=3.0, ρ=4.78, and $\theta_{e}=0.035$, which are utilized for all simulations unless stated otherwise.

## 4. Results and Discussion

### 4.1. Dynamic Characteristics of a Piezoelectric Energy Harvester Excited by a Single Plucking

**Figure 2:**
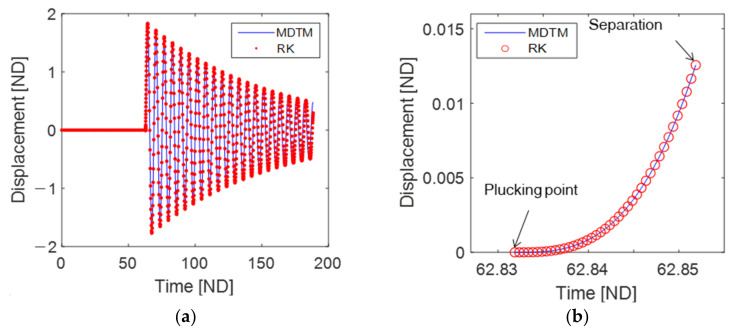
(**a**) Free vibration of the piezoelectric energy harvester when excited by a single plucking, with the parameter values of $w_{d}=0.6$ and γ=0.01, (**b**) a magnified view of the dynamic behavior in the contact phase. In both (**a**,**b**), the DTM solutions are represented by blue lines, while the numerical solutions obtained using the Runge–Kutta (RK) method are depicted as red dots or open circles. The initial conditions are set to zero, indicating that the system is at rest initially. The phase angle $\phi_{0}=0$ represent the position of the plectrum beneath the tip of the beam, indicating that the contact phase begins at τ=2π/ω=62.8 after one rotation cycle of the plectrum. The labels on the horizontal and vertical axes indicate non-dimensional quantities denoted by “ND”.

Figure 2 illustrates (a) the free vibration of the piezoelectric energy harvester excited by a single plucking event and (b) a magnified view of the contact phase during the mechanical plucking process. In this figure, the initial conditions of the energy harvesting oscillator are set to zero, namely, x(0)=0, $\dot{x}(0)=0$, and y(0)=0, ensuring that the oscillator is at rest before being plucked by a rotating plectrum. The initial angular position of the rotating plectrum is specified as $\phi_{0}=2\pi /3$. As the plectrum reaches a trivial position (i.e., u=x=0), it makes initial contact with the tip of the piezoelectric cantilever. Figure 2 presents a comparison between the MDTM solutions (blue lines) and the numerical solutions (red dots in Figure 2a or open circles in Figure 2b), which are computed using the Runge–Kutta (RK) method.

The plucking process of the piezoelectric energy harvester involves two distinct time scales of dynamic motion: (i) the contact phase between the cantilever and plectrum, which occurs for a very short duration, and (ii) the subsequent free vibration process of the piezoelectric cantilever, which generates electrical energy. Therefore, the MDTM solution procedures need to be applied separately to these two different time-scale motions.

The comparison between the MDTM solutions and the RK results in Figure 2 demonstrates strong agreement and confirms the validity of the analytic technique employed in this study. The analytical MDTM solution offers a simpler means of determining the duration of the contact phase compared to numerical methods. In Figure 2b, the starting and ending points of the contact phase correspond to the plucking and separation points, respectively. Since the piezoelectric cantilever is initially at rest, the plucking time $t_{p}$ can be determined by satisfying the condition of zero indentation at a trivial position, expressed as follows:

(23) $$t_{p}=\frac{2\pi -\phi_{0}}{\omega }\;\mathrm{at}\;u=w_{d}.$$

After the initial plucking event, the separation point can be determined by satisfying the conditions of zero indentation within the geometric contact zone ($0<u<w_{p}$). This can be expressed as follows:

(24) $$\tau_{s}=\left\{\begin{array}{cc} \tau :\;R\sin(\omega \tau +\phi_{0})-L^{-1}\left[P_{x}^{L/M}(s)\right](\tau )=0, & \mathrm{for}\;0<u\leq w_{d}\\ \frac{w_{d}}{R\omega }, & \mathrm{otherwise} \end{array}\right.,$$

where τ is the time defined as $\tau =t-t_{p}$ that is introduced for illustration purposes, so that the starting and ending points of the contact phase become at τ=0 and $\tau =\tau_{s}$, respectively.

**Figure 3:**
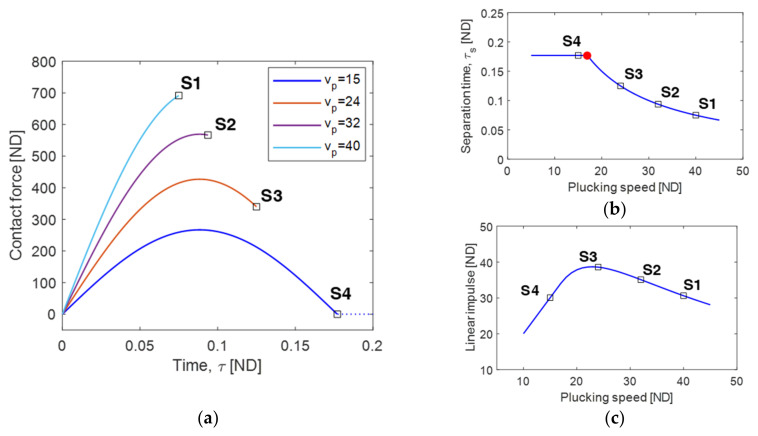
(**a**) Variations in contact force between the piezoelectric cantilever and plectrum during the contact phase, obtained for the plucking speeds of 15, 24, 32, and 40, (**b**) the variation in separation time with respect to the plucking speed, and (**c**) the variation in linear impulse. The horizontal axis is the time τ measured from the plucking time $t_{p}$, which is given as $\tau =t-t_{p}$, so that the starting point of the contact phase is located at the origin of τ=0. The open square points S1–S4 indicate the separation points. In (**a**), the dashed line denotes the loss of contact between the beam and the plectrum. In (**b**), the red solid circle point represents the critical condition of the plucking speed at which the separation time (or contact duration) is saturated and maximized.

In Figure 3a, the contact force between the piezoelectric cantilever and plectrum during the contact phase is depicted for different plucking speeds: 15, 24, 32, and 40. The ending points of the contact phase are indicated by the open square points S1–S4 corresponding to the respective plucking speeds.

As observed in Figure 3a, the duration of the contact phase, denoted as Δτ(=Δt), or the separation time, $\tau_{s}$, exhibits significant dependence on the plucking speed. When the plucking speed is relatively high, the contact phase duration or separation time is short, as the geometrical separation point is fixed at $u=w_{d}$. Consequently, as the plucking speed decreases, the separation time tends to increase, as evidenced by the open square points S1–S3 in Figure 3a,b.

However, this trend is interrupted at a critical point, indicated by the solid red circle in Figure 3b. This occurs because, at low plucking speeds, the relative motion of the cantilever beam can sufficiently induce the loss of contact, even before the plectrum reaches its geometrically designated separation distance. Moreover, it is interesting to note that the separation time reaches a saturation point as the plucking speed reaches a certain critical value. The saturation value of the separation time may be influenced by the rotational frequency of the plectrum rather than its plucking speed.

As discussed in Figure 3a, increasing the plucking speed leads to a shorter duration of the contact phase, but it also results in a higher maximum contact force. Consequently, the mechanical linear impulse does not exhibit a monotonic relationship with the plucking speed. Figure 3c presents the mechanical linear impulse of the contact force, integrated over the time interval of the contact phase, with respect to the plucking speed. This figure includes an optimal plucking speed that maximizes the linear impulse, thereby yielding the largest free vibration and highest output voltage for the piezoelectric energy harvester.

**Figure 4:**
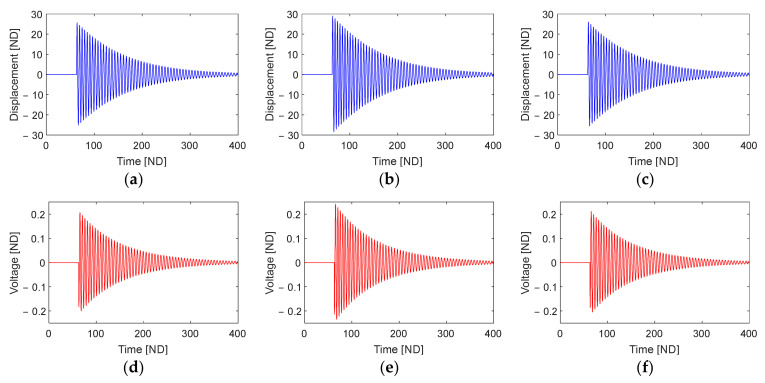

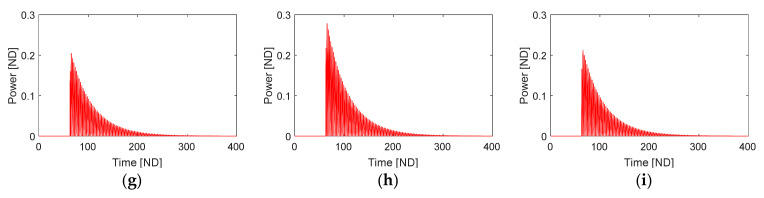
Time responses of the piezoelectric energy harvester excited by a single plucking, including the free vibrations (first row), output voltages (second row), and output powers (third row). These responses are obtained at three different points designated by S1 (**a**,**d**,**g**), S3 (**b**,**e**,**h**), and S4 (**c**,**f**,**i**) in Figure 3.

Figure 4 shows the time responses of the free vibration of the piezoelectric energy harvester and the corresponding output voltage at points S1, S3, and S4 (as shown in Figure 3). It is evident from the figure that the displacement obtained at point S3 is greater than those at points S1 and S4. The resulting RMS output voltages are 0.0484, 0.0572, and 0.0493 at points S1, S3, and S4, respectively.

**Figure 5:**
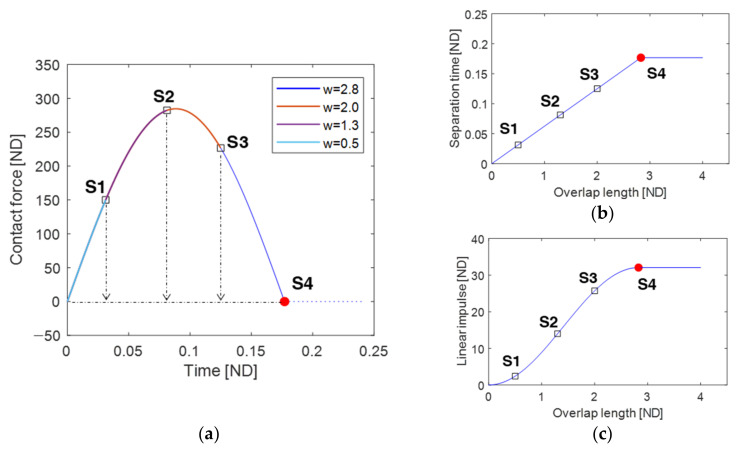
(**a**) Variations in contact force between the piezoelectric cantilever and plectrum during the contact phase, obtained for the overlap length of 0.5, 1.3, 2.0, and 2.8, and (**b**) the variation in separation time with respect to the overlap length and (**c**) the variation in l linear impulse. The open square points S1–S3 denote the separation points. Particularly, the red solid circle point S4 indicates the critical condition of maximum overlap length at which the separation time (or contact duration) and linear impulse are saturated and maximized.

Figure 5a displays the variations in contact force between the piezoelectric cantilever and plectrum during the contact phase. These variations are evaluated for different overlap lengths: 0.5, 1.3, 2.0, and 2.8. The overlap length plays a crucial role in determining the geometrical separation point in the contact phase, but it does not impact the dynamic interaction between the piezoelectric cantilever and plectrum. Therefore, as depicted in Figure 5a, the profiles of the contact force for the different overlap lengths are identical, except for the separation time.

As the overlap length increases, the geometrical separation point also increases proportionally. Consequently, when the overlap length is relatively small (or large), the time duration of the contact phase (or separation time) is also small (or large). The separation time tends to increase as the overlap length reaches the critical point S4, as observed in Figure 5a,b. The red circle point S4 represents the critical condition of the maximum overlap length, where the separation time and linear impulse reach their saturation points and are maximized, as shown in Figure 5b,c. As discussed in Figure 5a, as the overlap length increases, both the contact force and the time duration of the contact phase also increase. Consequently, the mechanical linear impulse tends to increase monotonically with the overlap length, as depicted in Figure 5c. Therefore, the critical point S4 indicates the optimal overlap length for maximizing the linear impulse and output voltage of the piezoelectric energy harvester. The effects of overlap length and plucking speed on the contact force and separation time in the plucking processes of the piezoelectric energy harvester have been recognized as important factors [38]. The analytical results presented in Figure 3 and Figure 5 are in good agreement with the numerical findings reported in the previous work [38], which further supports the validity of the analytical approach proposed in this study.

**Figure 6:**
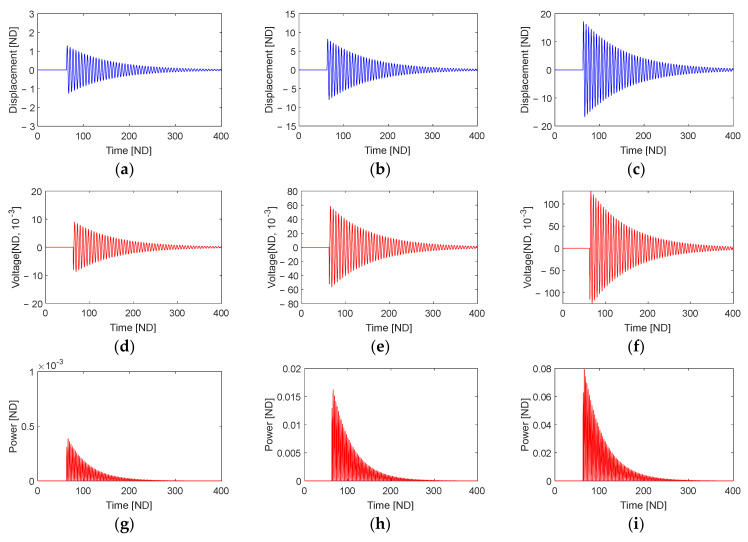
Time responses of the piezoelectric energy harvester excited by a single plucking, including the free vibrations (first row), output voltages (second row), and output powers (third row). These responses are obtained at three different points designated by S1 (**a**,**d**,**g**), S2 (**b**,**e**,**h**), and S3 (**c**,**f**,**i**) in Figure 5.

Figure 6 illustrates the time responses of the piezoelectric energy harvester’s free vibration and the corresponding output voltage evaluated at points S1, S2, and S3 (indicated in Figure 5). It is evident that the displacement of the free vibration during the release phase (Figure 6a–c) increases with the overlap length, and this trend is accompanied by a corresponding increase in the associated output voltages (Figure 6d–f) and output powers (Figure 6g–i). The calculated RMS output voltages are 0.0018, 0.0122, and 0.0287 at points S1, S2, and S3, respectively.

### 4.2. Periodic Plucking

In this section, we investigate the dynamic characteristics of the piezoelectric energy harvester when subjected to periodic plucking. The first plucking cycle corresponds to the free vibration of the energy harvester excited by a single plucking, as discussed in the previous section. Subsequent plucking cycles occur with a period determined by the rotational frequency of the plectrum. In the case where the piezoelectric cantilever is initially at rest, the plucking point for the first cycle is located at a trivial position. However, for subsequent cycles, the plucking point may vary if the free vibration of the piezoelectric cantilever is not fully damped out before the next plucking. Hence, the plucking time needs to be determined by solving the equation of zero indentation within the geometrical contact zone ($\left|u\right|\leq w_{p}$), as shown below:

(25) $$t_{p}=\left\{\begin{array}{cc} t:\;R\sin(\omega t+\phi_{0})-L^{-1}\left[P_{x}^{L/M}(s)\right](t)=0, & \mathrm{for}\;\left|u\right|\leq w_{d}\\ \;\mathrm{no}\;\mathrm{plucking}, & \mathrm{otherwise} \end{array}\right..$$

If a solution to Equation (25) cannot be found within the geometrical contact zone, it indicates that the plectrum does not make contact with the piezoelectric cantilever. If a single solution exists, it represents the plucking time that triggers the subsequent plucking cycle. In this case, the separation time is determined by the geometric separation point and can be evaluated as $\tau_{s}=w_{d}/R\omega$ presented in Equation (24). Lastly, if two distinct solutions are obtained, the first solution corresponds to the plucking time, while the second solution represents the separation time. This implies that the loss of contact between the piezoelectric cantilever and the plectrum occurs before the plectrum reaches the geometrically designated separation point.

**Figure 7:**
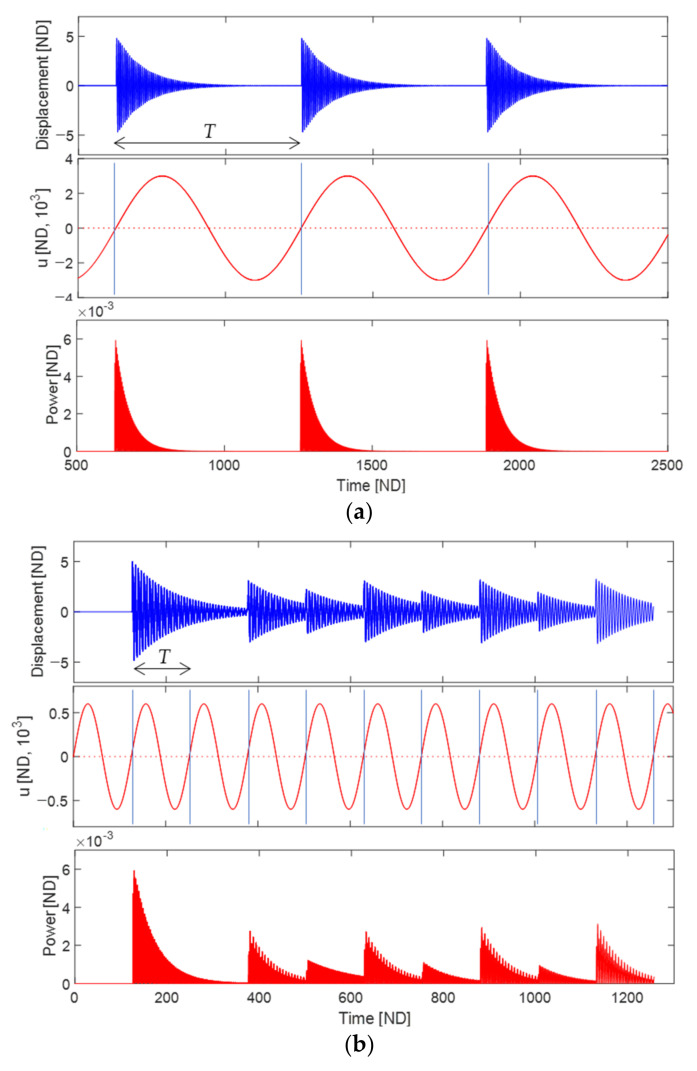
(**upper**) Free vibration of the piezoelectric energy harvester excited by periodic plucking, (**middle**) the rotational motion of the plectrum, and (**lower**) the corresponding output power. Two different rotational frequencies of the plectrum are considered: (**a**) 0.01 and (**b**) 0.05. In this figure, T denotes the plucking period.

Figure 7 illustrates the dynamic behavior of the piezoelectric energy harvester under periodic plucking. The upper part of the figure shows the free vibration of the harvester, while the middle part displays the corresponding rotational motion of the plectrum. The lower part of the figure represents the resulting output power. Two different rotational frequencies of the plectrum are considered: (a) 0.01 and (b) 0.05.

In Figure 7a, the plucking period given as T=628.3 is relatively long. As a result, the free vibration of the piezoelectric beam is almost damped out before the beam is plucked at rest by the plectrum. Therefore, it appears that the free vibration excited by a single plucking cycle (as discussed in the previous section) is repeated in each plucking cycle. However, from the perspective of energy harvesting efficiency, it would be more desirable to minimize the portion of small-amplitude free vibration within each plucking cycle. This can be achieved by reducing the plucking period. 

In Figure 7b, the plucking period is given as T=125.7. When comparing Figure 7a,b, it can be observed that as the rotational frequency of the plectrum increases, the plucking period becomes shorter, although the dynamic interaction during the contact phase becomes more complex. The first free vibration appears to be relatively larger than the subsequent ones because the relative motion of the cantilever beam in the subsequent plucking cycles potentially reduces the contact force magnitude due to decreased indentation of the contact surface. However, despite this observation, the RMS output voltage in Figure 7b is still higher than that in Figure 7a. Additionally, it is noteworthy in Figure 7b that the plectrum fails to pluck the piezoelectric cantilever in the second cycle. While a shorter plucking period may improve energy harvesting efficiency if the free vibration is successfully triggered in every plucking cycle, it often leads to the failure of the plectrum in periodic plucking.

**Figure 8:**
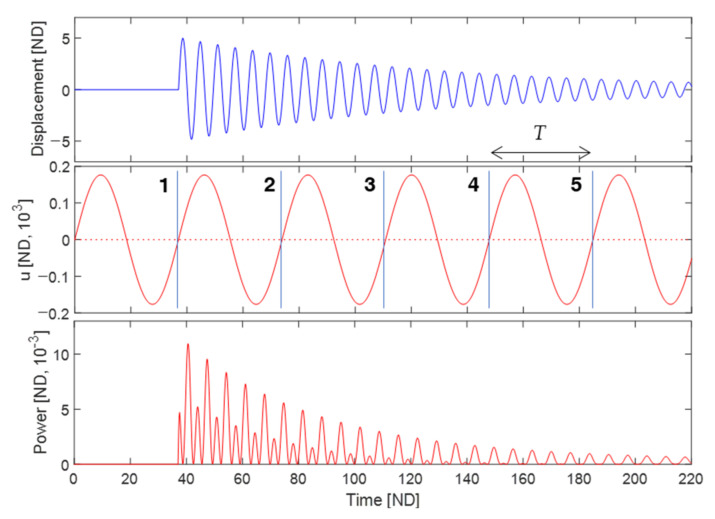
(**upper**) Free vibration of the piezoelectric energy harvester excited by periodic plucking, (**middle**) the rotational motion of the plectrum, and (**lower**) the corresponding output power, obtained when the rotational frequency of the plectrum is 0.17.

Figure 8 shows an example of continuous plectrum failure in periodic plucking, obtained with a plucking frequency of ω=0.17. In this simulation, the plectrum successfully plucks the piezoelectric cantilever at rest only during the first plucking cycle, while subsequently failing to do so for four consecutive cycles. The intentional choice of the rotational frequency in this case causes a lack of synchronization between the motions of the piezoelectric cantilever and the plectrum within their geometrically designated contact zone. In contrast, the plucking frequencies used in Figure 7a,b are specifically chosen to ensure exact synchronization:

(26) $$\omega_{n}=m\omega \;\mathrm{or}\;T=m\tau_{n},\;m=1,2,3,\cdots$$

where $\omega_{n}$ and $\tau_{n}$ are the natural angular frequency and natural period of the piezoelectric cantilever oscillator, respectively, which are given by $\omega_{n}=1$ and $\tau_{n}=2\pi$ in this study, and m is the positive integer. Equation (26) indicates that the reciprocal of the frequency ratio, $\omega_{n}/\omega$, must be a positive integer to achieve an exact synchronization between two periodic motions. This condition is essential as a necessary requirement and a fundamental design strategy, even though nonlinear effects also need to be considered. In Figure 7a,b, the inverse frequency ratios used are 100 and 20, respectively, both of which are positive integers. However, in Figure 8, the inverse value is not an integer but rather 5.88. It should be noted that reducing the plucking speed of the plectrum and increasing the overlap length can offer further advantages in the context of periodic-plucking energy harvesting.

## 5. Conclusions

In this study, we proposed an analytical approach based on the modified differential transform method to investigate the dynamic behavior of a mechanical plucking energy harvester. The energy harvester consisted of a piezoelectric cantilever oscillator and a rotating plectrum. Our analytical approach provided a closed-form solution for the beam displacement, allowing us to determine the starting and ending points of the contact phase between the piezoelectric cantilever and the plectrum, as evaluated by Equations (24) and (25). This approach proved valuable in simulating complex dynamic interferences during multiple or periodic plucking processes, where the contact phase varied depending on the relative motion of the beam.

To evaluate the effects of plucking speed and overlap length of the plectrum on single or periodic plucking, we conducted a series of simulations. Our observations showed that the output voltage of the piezoelectric energy harvester increased with the overlap length of the plectrum. This was because a longer contact duration resulted in a stronger linear impulse, triggering larger-amplitude free vibrations of the piezoelectric oscillator. On the other hand, increasing the plucking speed of the plectrum tended to enhance the magnitude of the contact force but shorten the contact phase duration. Therefore, optimizing the plucking speed was crucial to maximize the resulting linear impulse, leading to higher electric energy production from the piezoelectric cantilever.

For successful periodic plucking, it was essential to synchronize the motions of the piezoelectric energy harvester and the rotating plectrum within a limited contact zone. This synchronization could be achieved by matching the rotating frequency of the plectrum with an integer multiple of the natural frequency of the energy harvester. Failure to achieve this synchronization often resulted in the plectrum missing the piezoelectric cantilever in the contact zone due to dynamic interferences. Additionally, reducing the plucking speed of the plectrum and increasing the overlap length were advantageous for successful periodic-plucking energy harvesting.

It is important to note that the Duffing-type oscillator model used in this study represents a simplified approach to simulate the complex dynamics involved in multiple plucking scenarios of a piezoelectric energy harvester. Therefore, detailed design factors such as the geometric configurations of the rotational plectrum and the nonlinear curvature of the beam were not considered in the model. Further research, encompassing both experimental and theoretical studies, is necessary to gain a deeper understanding of the intricate plucking dynamics involved in piezoelectric energy harvesters.

## Figures and Tables

**Table 1:** Geometric parameters and material properties of a piezoelectric energy harvester.

Parameter	Value	
	Substrate	Piezoelectric layers (PSI-5H5E)
Length	48.8 mm	13.2 mm
Width	12.7 mm	12.7 mm
Thickness	0.60 mm	0.277 mm
Density	7640 kg/m^3^	7520 kg/m^3^
Young’s modulus	167 GPa	46 GPa
Piezoelectric constant $e_{31}$	-	−10.8 C/m^2^
Permittivity constant $\varepsilon_{33}^{S}$	-	25.1 nF/m

**Table 2:** Fundamental operations of the differential transform method.

Original Function	Transformed Function
u(t)	$U(k)=\frac{1}{k!}{\left.\frac{d^{k}f\left(t\right)}{dt^{k}}\right|}_{t=t_{0}}$
αu(t)±βv(t)	αU(k)±βV(k)
$t^{r}$	$\delta_{kr}$ (Kronecker Delta)
u(t)v(t)	$\sum_{r=0}^{k}U(r)V(k-r)$
u(t)v(t)w(t)	$\sum_{k_{2}=0}^{k}\sum_{k_{1}=0}^{k_{2}}U(k_{1})V(k_{2}-k_{1})W(k-k_{2})$
$\frac{d^{n}u(t)}{dt^{n}}$	$\frac{(k+n)!}{k!}U(k+n)$
sinωt	$\frac{\omega^{k}}{k!}\sin\left(\omega t_{0}+\frac{k\pi }{2}\right)$
cosωt	$\frac{\omega^{k}}{k!}\cos\left(\omega t_{0}+\frac{k\pi }{2}\right)$

**Figure A1:**
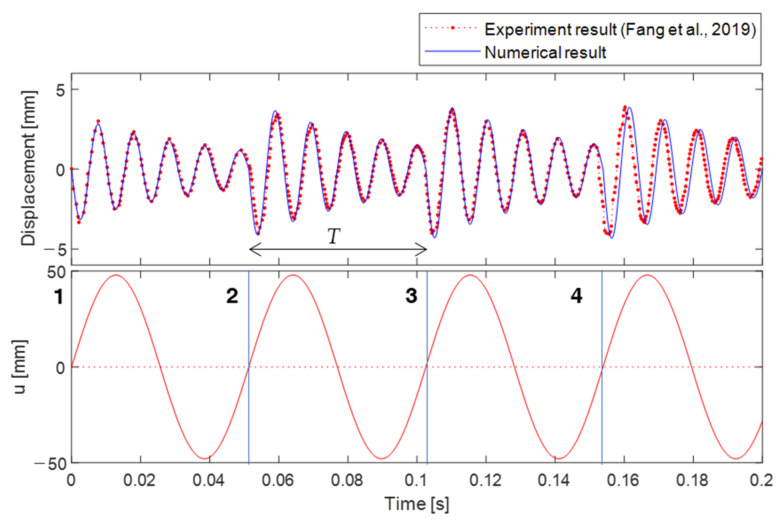
(**upper**) Free vibration of the piezoelectric energy harvester excited by periodic plucking, and (**lower**) the associated rotational motion of the plectrum. The numerical result (blue solid line) is directly compared with the experimental result (red dotted line) reported in Ref. [37].

## Data Availability

Not applicable.

## Appendix A

For a thin beam (based on the Euler–Bernoulli beam theory), the piezoelectric constitutive equations for the stress and electric displacement components are given as

(A1) $$T_{1}=c_{11}^{E}S_{1}+e_{31}E_{3},\;D_{3}=e_{31}S_{1}+\varepsilon_{33}^{S}E_{3},$$

where $T_{1}$ and $D_{3}$ are the stress and electric displacement components, respectively, $S_{1}$ and $E_{3}$ are the strain and electric field components, respectively, $c_{11}^{E}$, $e_{31}$, and $\varepsilon_{33}^{S}$ are the elastic, piezoelectric, and permittivity constants, respectively, and the superscript E and S denote that the respective constants are evaluated at the constant electric field and constant strain. In Equation (A1), the strain component can be expressed as

(A2) $$S_{1}=-\frac{h_{p}+h_{s}}{2}\kappa (s,t),$$

where $h_{p}$ and $h_{s}$ are the thicknesses of the piezoelectric layer and substrate, respectively, and κ is the curvature. The electric current output can be obtained from the integral form of Gauss’s law:

(A3) $$\frac{d}{dt}\left(\int_{A}D\cdot n\;dA\;\right)=\frac{\tilde{V}}{R},\;$$

where **D** is the electric displacement vector, **n** is the unit outward normal to the electrode area *A*, and $\tilde{V}$ is the output voltage across the resistance *R*. Substituting Equations (A1) and (A2) into Equation (A3), the equivalent circuit equation for the piezoelectric layers can be obtained as

(A4) $$C_{p}\frac{d\tilde{V}}{dt}+\frac{1}{R}\tilde{V}+\theta \frac{\partial \tilde{w}}{\partial t}=0,$$

where $\tilde{w}$ is the tip deflection of the piezoelectric beam, and $C_{p}$ and θ are the equivalent capacitance and electromechanical coupling constant, which are given as

(A5) $$C_{p}=\frac{\varepsilon_{33}^{S}b\;L_{p}}{2h_{p}},\;\theta =\frac{e_{31}b\;}{\phi (L)}\frac{h_{p}+h_{s}}{2}\int_{0}^{L_{p}}\left(\frac{d^{2}\phi (s)}{ds^{2}}\right)\;ds,$$

where b and $L_{p}$ are the width and length of the piezoelectric layer, respectively, and ϕ is the mode shape function. Note that the above derivation process was based on the single-mode approximation.

## Appendix B

To validate the oscillator model, a comparison is made between the numerical result obtained using Equations (1) and (2) and the experimental one reported in Ref. [37]. To this end, the system parameters of the oscillator model are evaluated using the geometric parameters and material properties of a piezoelectric energy harvester described in Ref. [37], which are listed in Table A1. The transient response of the piezoelectric energy harvester is obtained through direct numerical integration of a linear system of state equations using the Runge–Kutta method implemented with the ode45 function in MATLAB.

**Table A1:** Geometric parameters and material properties of a piezoelectric energy harvester used in Ref. [37].

Parameter	Value	
	Substrate	Piezoelectric layers
Length	56.8 mm	46.4 mm
Width	10.5 mm	6.5 mm
Thickness	0.6 mm	0.2 mm
Density	8363 kg/m^3^	7488 kg/m^3^
Young’s modulus	75 GPa	40 GPa
Piezoelectric constant $d_{31}$	-	−186 pm/V
Permittivity constant $\varepsilon_{33}^{S}$	-	19.52 nF/m

Figure A1 shows (upper) the free vibration of the piezoelectric energy harvester excited by periodic plucking and (lower) the corresponding rotational motion of the plectrum. It is worth noting that the plectrum rotates in a counterclockwise direction, while the plucking frequency and plucking period are given as 19.5 Hz and 50.3 ms, respectively. Figure A1 presents four plucking processes with the period of T, followed by the subsequent free vibrations. The numerical result obtained in this study exhibits good agreement with the experimental result reported in Ref. [37], which supports the effectiveness of employing the oscillator model to simulate the intricate dynamics involved in multiple plucking scenarios of a piezoelectric energy harvester.

## References

[1] Cheng Y., Wu N., Wang Q. (2017). An efficient piezoelectric energy harvester with frequency self-tuning. J. Sound Vib..

[2] Pennisi G., Mann B., Naclerio N., Stephan C., Michon G. (2018). Design and experimental study of a Nonlinear Energy Sink coupled to an electromagnetic energy harvester. J. Sound Vib..

[3] Le C.P., Halvorsen E., Søråsen O., Yeatman E.M. (2012). Microscale electrostatic energy harvester using internal impacts. J. Intell. Mater. Syst. Struct..

[4] Mann B.P., Sims N.D. (2009). Energy harvesting from the nonlinear oscillations of magnetic levitation. J. Sound Vib..

[5] Roundy S., Wright P.K., Rabaey J. (2003). A study of low level vibrations as a power source for wireless sensor nodes. Comput. Commun..

[6] Abdelkefi A., Najar F., Nayfeh A.H., Ben Ayed S. (2011). An energy harvester using piezoelectric cantilever beams undergoing coupled bending–torsion vibrations. Smart Mater. Struct..

[7] Wu H., Tang L., Yang Y., Soh C.K. (2012). A novel two-degrees-of-freedom piezoelectric energy harvester. J. Intell. Mater. Syst. Struct..

[8] Shahruz S.M. (2006). Limits of performance of mechanical band-pass filters used in energy scavenging. J. Sound Vib..

[9] Zhu D., Roberts S., Tudor M.J., Beeby S.P. (2010). Design and experimental characterization of a tunable vibration-based electromagnetic micro-generator. Sens. Actuators A Phys..

[10] Ibrahim S.W., Ali W.G. (2012). A review on frequency tuning methods for piezoelectric energy harvesting systems. J. Renew. Sustain. Energy.

[11] Stanton S.C., Erturk A., Mann B.P., Inman D.J. (2010). Nonlinear piezoelectricity in electroelastic energy harvesters: Modeling and experimental identification. J. Appl. Phys..

[12] Abdelkefi A., Nayfeh A.H., Hajj M.R. (2012). Global nonlinear distributed-parameter model of parametrically excited piezoelectric energy harvesters. Nonlinear Dyn..

[13] Kim P., Yoon Y.-J., Seok J. (2016). Nonlinear dynamic analyses on a magnetopiezoelastic energy harvester with reversible hysteresis. Nonlinear Dyn..

[14] Cottone F., Vocca H., Gammaitoni L. (2009). Nonlinear energy harvesting. Phys. Rev. Lett..

[15] Stanton S.C., McGehee C.C., Mann B.P. (2010). Nonlinear Dynamics for broadband energy harvesting: Investigation of a bistable piezoelectric inertial generator. Phys. D Nonlinear Phenom..

[16] Erturk A., Inman D.J. (2011). Broadband piezoelectric power generation on high-energy orbits of the bistable Duffing oscillator with electromechanical coupling. J. Sound Vib..

[17] Daqaq M.F., Masana R., Erturk A., Quinn D.D. (2014). On the role of nonlinearities in vibratory energy harvesting: A critical review and discussion. Appl. Mech. Rev..

[18] Kim P., Seok J. (2014). A multi-stable energy harvester: Dynamic modeling and bifurcation analysis. J. Sound Vib..

[19] Cao J., Inman D.J., Lin J., Liu S., Wang Z. (2014). Broadband tristable energy harvester: Modeling and experiment verification. Appl. Energy.

[20] Kim P., Seok J. (2015). Dynamic and energetic characteristics of a tri-stable magnetopiezoelastic energy harvester. Mech. Mach. Theory.

[21] Cao J., Zhou S., Wang W., Lin J. (2015). Influence of potential well depth on nonlinear tristable energy harvesting. Appl. Phys. Lett..

[22] Nguyen M.S., Yoon Y.-J., Kwon O., Kim P. (2017). Lowering the potential barrier of a bistable energy harvester with mechanically rectified motion of an auxiliary magnet oscillator. Appl. Phys. Lett..

[23] Noh J., Nguyen M.S., Kim P., Yoon Y.-J. (2021). Load Resistance Optimization of a Magnetically Coupled Two-Degree-of-Freedom Bistable Energy Harvester Considering Third-Harmonic Distortion in Forced Oscillation. Sensors.

[24] Noh J., Nguyen M.S., Kim P., Yoon Y.-J. (2022). Harmonic balance analysis of magnetically coupled two-degree-of-freedom bistable energy harvesters. Sci. Rep..

[25] Renaud M., Fiorini P., van Schaijk R., Van Hoof C. (2009). Harvesting energy from the motion of human limbs: The design and analysis of an impact-based piezoelectric generator. Smart Mater. Struct..

[26] Gu L., Livermore C. (2011). Impact-driven, frequency up-converting coupled vibration energy harvesting device for low frequency operation. Smart Mater. Struct..

[27] Fu X., Liao W.H. (2018). Nondimensional model and parametric studies of impact piezoelectric energy harvesting with dissipation. J. Sound Vib..

[28] Priya S., Chen C.T., Fye D., Zahnd J. (2004). Piezoelectric windmill: A novel solution to remote sensing. Jpn. J. Appl. Phys..

[29] Toma D.M., del Rio J., Carbonell-Ventura M., Masalles J.M. Underwater energy harvesting system based on plucked-driven piezoelectrics. Proceedings of the OCEANS 2015—Genova.

[30] Kuang Y., Zhu M. (2017). Design study of a mechanically plucked piezoelectric energy harvester using validated finite element modelling. Sens. Actuators A Phys..

[31] Priya S. (2005). Modeling of electric energy harvesting using piezoelectric windmill. Appl. Phys. Lett..

[32] Pozzi M., Zhu M. (2011). Plucked piezoelectric bimorphs for knee-joint energy harvesting: Modeling and experimental validation. Smart Mater. Struct..

[33] Pozzi M., Zhu M. (2012). Characterization of a rotary piezoelectric energy harvester based on plucking excitation for knee-joint wearable applications. Smart Mater. Struct..

[34] Bai F., Song G., Dong W., Guan L., Bao H. (2017). Fan-structure wind energy harvester using circular array of polyvinylidene fluoride cantilevers. J. Intell. Mater. Syst. Struct..

[35] Pozzi M. (2018). Synchronicity and pure bending of bimorphs: A new approach to piezoelectric energy harvesting. Smart Mater. Struct..

[36] Fang S., Fu X., Liao W.H. (2019). Analysis of the interference in typical rotational plucking energy harvester. Active and Passive Smart Structures and Integrated Systems XII.

[37] Fang S., Fu X., Liao W.H. (2019). Modeling and experimental validation on the interference of mechanical plucking energy harvesting. Mech. Syst. Signal Process..

[38] Fu X., Liao W.H. (2019). Modeling and Analysis of Piezoelectric Energy Harvesting with Dynamic Plucking Mechanism. J. Vib. Acoust..

[39] Fang S., Fu X., Liao W.H. (2019). Asymmetric plucking bistable energy harvester: Modeling and experimental validation. J. Sound Vib..

[40] Zhou J.K. (1986). Differential Transformation and Its Applications for Electrical Circuits.

[41] Keskin Y., Kurnaz A., Kiris M., Oturanc G. (2007). Approximate solutions of generalized pantograph equations by the differential transform method. Int. J. Nonlinear Sci. Numer. Simul..

[42] Baker G.A. (1975). Essentials of Padé Approximants.

[43] Benhammouda B., Vazquez-Leal H., Sarmiento-Reyes A. (2014). Modified reduced differential transform method for partial differential algebraic equations. J. Appl. Math..

